# Marine reserves lag behind wilderness in the conservation of key functional roles

**DOI:** 10.1038/ncomms12000

**Published:** 2016-06-29

**Authors:** Stéphanie D'agata, David Mouillot, Laurent Wantiez, Alan M. Friedlander, Michel Kulbicki, Laurent Vigliola

**Affiliations:** 1 MARBEC, UMR IRD-CNRS-UM-IFREMER 9190, Université Montpellier, Languedoc-Roussillon, 34095 Montpellier Cedex, France; 2 ENTROPIE, UMR IRD-UR-CNRS 9220, Laboratoire d'Excellence LABEX CORAIL, Institut de Recherche pour le Développement, BP A5, 98848 Nouméa Cedex, New Caledonia; 3 Wildlife Conservation Society, Global Marine Program, Bronx, New York 10460 USA; 4 Université de Nouvelle Calédonie-EA4243 Laboratoire « LIVE » – BP R4, 98851 Nouméa-Nouvelle Calédonie; 5 Fisheries Ecology Research Lab, University of Hawaii, 2538 McCarthy Mall, Honolulu, Hawaii 96822, USA; 6 Pristine Seas-National Geographic Society, Washington, DC 20036, USA; 7 ENTROPIE, UMR IRD-UR-CNRS 9220, Laboratoire d'Excellence LABEX CORAIL, Institut de Recherche pour le Développement, Université de Perpignan, 66860 Perpignan Cedex 9, France

## Abstract

Although marine reserves represent one of the most effective management responses to human impacts, their capacity to sustain the same diversity of species, functional roles and biomass of reef fishes as wilderness areas remains questionable, in particular in regions with deep and long-lasting human footprints. Here we show that fish functional diversity and biomass of top predators are significantly higher on coral reefs located at more than 20 h travel time from the main market compared with even the oldest (38 years old), largest (17,500 ha) and most restrictive (no entry) marine reserve in New Caledonia (South-Western Pacific). We further demonstrate that wilderness areas support unique ecological values with no equivalency as one gets closer to humans, even in large and well-managed marine reserves. Wilderness areas may therefore serve as benchmarks for management effectiveness and act as the last refuges for the most vulnerable functional roles.

## Abstract (web summary)

The establishment of marine reserves in areas previously exploited by humans is recognised as an effective conservation tool. Through modelling effects of isolation from human influence, D'agata *et al*. show that marine reserves fall short of supporting key ecological values compared with wilderness areas.

## Graphical abstract

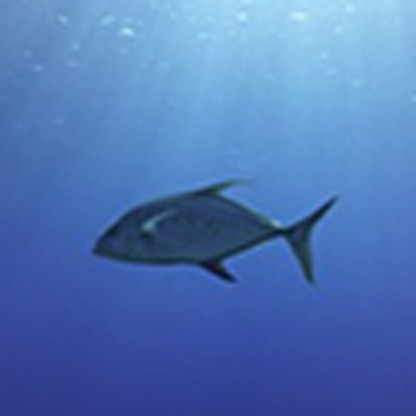

The establishment of protected areas for cultural and resource purposes is longstanding and universal in the history of humankind^1^, with current global coverage of 12.7% on land but only 7.2% for the marine realm^2^. Marine reserves, that is, no-take Marine Protected Areas (MPAs)^3^, are widely recognized as effective conservation tools supporting greater biodiversity and biomass, up to several orders of magnitude, than nearby exploited areas^4^ and providing socio-economic benefits, even beyond their boundaries^2^,^3^,^5^,^6^. However, most marine reserves are embedded in regions with deep and long-lasting human footprints, or are typically established in response to degradation^7^. Thus, even the best marine reserves, that is, those cumulating key features^4^ (old, large and no entry), may only partially restore impacted ecosystems or provide limited benefits and may thus underestimate conservation targets to measure management effectiveness.

Pre-human impact should be considered as a baseline for assessing degradation and restoration effectivness^8^,^9^. Yet, for some taxa such as fishes, past data are limited regarding biomass and other key metrics such as functional diversity that represents the breadth of roles played by species on which ecosystem functioning depends^10^. As an alternative, the last wilderness areas in the ocean, far from human influences, may provide the most accurate baselines^7^,^11^ to assess both the level of degradation and marine reserve restoration effectiveness. However, wilderness areas, owing to their isolation, may possess particular fauna, habitats and environments that may bias comparisons with distant marine reserves or exploited areas. Thus, the extent to which marine reserves can achieve the same ecological value as wilderness areas remains unknown, in particular for critical aspects that regulate the functioning of coral reef ecosystems such as the biomass of top predators^12^ and herbivores^13^,^14^ or the level of functional diversity^15^.

New Caledonia (South-Western Pacific) offers a unique opportunity to test whether marine reserves can achieve the same ecological value as wilderness areas, as, at a regional scale and for a given species pool, there is (i) a strong gradient of human influence from the capital city market of the island (Noumea, 98,000 inhabitants), (ii) a large variety of restrictions ranging from traditionally managed areas to long established (up to 38 years) and large (up to 175 km^2^) no-entry marine reserves, and (iii) extensive surveys (1,833 underwater visual censuses) of fish communities on coral reefs (Fig. 1). Here we modelled the ‘pure' effect of isolation from human influence, through the travel time from the market^16^,^17^, for six complementary metrics of fish community structure (total biomass, biomass of top predators and herbivores, species richness, functional richness and biomass-weighted functional diversity) by decoupling the influence of confounding natural variables (Methods). We then characterized the shape (saturating or not and potential thresholds) of these modelled relationships to delimit wilderness areas, if any, in New Caledonia. Finally, we tested whether marine reserves can reach the same ecological value as wilderness areas or whether some particular functional roles are lacking. We show that the levels of the six fish community metrics only saturate beyond a threshold of 20 h travel time from the market. In comparison with those wilderness areas, even the most effective marine reserve has 3.5 times lower biomass of apex predators and misses some key functional roles such as mobile top predators.

## Results

### Drivers of fish community structure

Using 11 explanatory variables related to coral reef environment, geography, geomorphology, habitat and human influence, boosted regression tree (BRT) models, which are able to cope with interactions and nonlinear relationships^18^, explained between 40 and 70% of the variance (cross-validation, CV) in the 6 fish community metrics across the 1,626 transects surveyed outside marine reserves (Supplementary Table 1, 15–49% of explained deviance). The relative contributions of these explanatory variables (Supplementary Figs 1 and 2) revealed that human influence, determined here by travel time from the market, was strongly associated with all community metrics, in particular functional richness (44%), that is, the breadth of fish functional roles (Methods). This result points out that, in the absence of fishing restrictions, the isolation of coral reefs from humans and market influence is primarily shaping fish community structure beyond total biomass alone^16^,^17^.

Species richness was also influenced by live coral cover and depth, confirming the importance of habitat characteristics for the diversity of fishes hosted by coral reefs^19^,^20^,^21^. Beyond isolation, total biomass and biomass of herbivores were also driven by sea surface temperature (SST) in accordance with metabolic theory that predicts an increase in the metabolic rate of organisms with increasing temperature, especially for herbivores^22^,^23^. The directions of the relationships between all environmental variables and each of the six fish community metrics are presented in Supplementary Table 2.

### Effect of isolation

To better examine how isolation shapes fish community structure, we extracted the ‘pure' marginal effect of travel time from the market on each community aspect and selected the best model representing each relationship (Methods). The sigmoid model was consistently selected (Supplementary Table 3), highlighting that all community metrics increased nonlinearly with increasing isolation until reaching a saturating plateau after 20 h travel time from the market, which we considered as delimitation for wilderness in New Caledonia (Fig. 2).

Top predator biomass and functional richness showed the steepest increase (69.3 and 60.6% differences, respectively) along the entire gradient of isolation (that is, from areas close to the market towards wilderness areas). In comparison, species richness and biomass-weighted functional diversity were weakly influenced by isolation, with a difference of 16.9 and 10.5%, respectively. The inflexion point on the sigmoid curves, marking the maximum increase, varied depending on the community aspect; biomass rose faster (6.5 h for total fish biomass) than biodiversity (21.5 h for fish functional diversity) along the isolation gradient. This highlights that key ecological functions are still missing even after total biomass reaches its maximum.

Although care should be taken in generalizing these thresholds to define wilderness, they may serve as a guide for other developed countries with strong fishing pressure in the water-surrounding populated areas, yet with well enforced restrictions on their exclusive economic zone and remote reefs. Travel time from the market is also somehow integrating local human density, as, in our case, they are closely related, albeit only between 0 and 9 h travel time whatever the buffer size (between 10 and 100 km) considered to estimate population density (Supplementary Fig. 3a,c). This implies that fish biomass and biodiversity are certainly impacted by both local human population and economic drivers such as proximity to market. However, their relative contributions are difficult to disentangle within the range of 9 h of travel time in our study. Yet, after a certain degree of isolation, distal human pressure such as the influence of the main market is still present even if there is no local human population surrounding the reef. This explains our choice to consider travel time from the market as a more continuous and integrative proxy of human pressure than human density^17^.

### Comparisons along a large human gradient

We then compared the levels of the six fish community metrics among six categories of areas as follows: (i) the best marine reserve (38 years old, no entry, large with 17,500 ha and well enforced); (ii) small marine reserves (<5 km^2^); (iii) traditionally managed areas <5 h travel time; (iv) isolated traditionally managed areas (19 h travel time); (v) exploited areas (<3 h travel); and (vi) wilderness areas (>20 h travel) (Fig. 3). The different categories had significantly different levels of fish biomass and biodiversity (*P<*0.001) (Supplementary Table 4). The biomass of top predators showed the most remarkable difference among categories, being 3.5 times greater in wilderness areas (median, 35 g m^−2^) compared with any other area, including the best marine reserve (median, 10 g m^−2^), even when controlling for environmental, geographic, geomorphologic and habitat variables (Supplementary Figs 4 and 5, and Supplementary Table 4). Total biomass and biomass of herbivores were also greater in wilderness areas than in the best marine reserve, albeit not significantly different (Fig. 3a,c). Fish biomass, and in particular that of top predators, in the wilderness areas of New Caledonia was similar or higher to those previously reported in the most isolated Indo-Pacific reefs (Chagos Archipelago^11^, Hawaiian Archipelago^24^, Cocos Island^25^ or Line Islands^26^), supporting these baseline levels at least in the Indo-Pacific.

Functional richness was not significantly different between wilderness and isolated traditionally managed areas (Fig. 3d). The levels of species richness and biomass-weighted functional diversity were very similar between wilderness areas, marine reserves and isolated traditionally managed areas (Fig. 3b,f). At the opposite end of the gradient, small marine reserves showed higher levels for all fish community metrics than exploited or small traditionally managed areas (<5 h), albeit nonsignificantly. Small marine reserves close to the market never achieved the same ecological value as isolated areas for any fish community metric. In contrast, the large and isolated traditionally managed areas (19 h) showed comparable levels of fish community structure to wilderness, except for the biomass of top predators, which was significantly higher in the latter category (Fig. 3e).

The levels of fish biomass and biodiversity within marine reserves are often related to their size, age, restrictions and isolation from human populations^4^,^27^. Larger marine reserves tend to better protect fishes with large home ranges, but ecological recovery after strict closure to fishing can range from 20 to 40 years^28^,^29^. Here, the best marine reserve, possessing four out of five key features for conservation effectiveness (old, large, enforced and no entry)^4^, should be considered close to a historical unfished baseline. The level of biomass in this marine reserve (around 1,200 kg ha^−1^) is comparable to other highly effective marine reserves in coral reef ecosystems^11^. Yet, its fish functional richness, although being higher than in smaller and younger marine reserves, still lags behind the level observed in large and isolated traditionally managed areas or in wilderness areas, with some key functional groups absent. In particular, the biomass of top predators in the best marine reserve is less than half that observed in wilderness areas (Fig. 3e) even after controlling for confounding variables (Supplementary Figs 4 and 5). Conversely, this best marine reserve and the large, isolated and traditionally managed areas are similar to wilderness areas for conserving biomass of other functional groups such as herbivores, which may play an essential role in the prevention of phase shifts from coral to algal-dominated states^13^,^14^. Herbivore biomass is low in fished areas, as they are heavily targeted by fishers in New Caledonia^30^. Smaller marine reserves only reach 28 and 27% of herbivore biomass found in wilderness areas and in the best reserve, respectively (Fig. 3c).

### Missing fish functional roles in MPAs

To highlight which fish functional roles are missing in exploited areas, marine reserves and traditionally managed areas compared with wilderness areas, we built a two-dimensional functional space to examine species according to their ecological traits^31^ (Fig. 4 and Supplementary Fig. 6). Fish communities in wilderness areas filled a larger volume, corresponding to a larger breadth of functional roles, than fish communities in marine reserves, traditionally managed areas or exploited areas. The common volume filled by at least 50% of the fish communities, representing the breadth of core functions present in a given area, drastically increased (from 10 to 76%) (Supplementary Table 5) with isolation from the market, suggesting that many functional roles are consistently missing in most fish communities, even in the best marine reserve. For example, mobile top predators, such as jacks, were only common in large, isolated and traditionally managed areas and wilderness areas (Fig. 4d,g). The proximity of marine reserves to highly populated places (<3 h of travel time) and the absence of deep geographical barrier (that is, sandy areas >25 m depth), which may act as a natural protection against fishing^4^, might explain the absence of mobile top predators in the largest and oldest marine reserve compared with wilderness areas. These fishes are highly targeted outside marine reserves and probably need large areas to sustain healthy populations^32^. High fishing pressure close to populated places can have negative effects within even well-enforced marine reserves. Conversely, large invertivores and sedentary top predators (for example, large groupers) were common in wilderness areas but also in the best marine reserve and isolated traditionally managed areas. This suggests that most of crucial ecosystem functions can be maintained through a range of fisheries restrictions^33^.

## Discussion

The contrasts observed between wilderness areas and fished areas or marine reserves are probably conservative and may be even more pronounced in the developing world where MPAs have fewer key features (for example, fewer restriction and degraded habitats)^4^,^34^ and where fishing pressure is higher^35^. In addition, our results are conservative, as wilderness areas of New Caledonia were sampled between 2012 and 2013, whereas most of exploited areas were sampled in the 1980s and 1990s. As human impacts tend to increase through time in all ecosystems across the world (∼57% population growth between 1986 and 2013 in New Caledonia, mainly at Noumea), we suggest that levels of fish biodiversity and biomass in the past were at least as high as those observed in 2013 in wilderness areas and certainly lower in 2013 than in the past for exploited areas. Thus, the observed contrasts in our results would have been even more pronounced if all fish communities were sampled synchronously in 2013.

By examining the structure of fish communities along a gradient of isolation and protection, we highlight the nested and complementary effectiveness of wilderness, marine reserves and traditionally managed areas in conserving the breadth of functional roles played by fishes on coral reefs. Old and large marine reserves can be effective in sustaining high biomass of key groups such as herbivores, but wilderness areas are essential for preserving viable populations of large mobile predators that support unique roles in the functioning of marine ecosystems through trophic regulation and nutrient transfer across habitats^36^. Traditionally managed areas, if sufficiently large and isolated, can also sustain biomass and biodiversity levels comparable to wilderness areas. Small marine reserves in highly populated areas can only sustain a small fraction of the whole fish community present in wilderness areas. However, these small no-take marine reserves located in human-dominated areas can provide recreational and educational benefits^37^, and can also serve as seedling cradles to enhance population recovery after disturbance^38^.

Wilderness areas are too rare on earth to replace marine reserves but should be considered as benchmarks, after controlling for confounding variables, for measuring management effectiveness and as the last sanctuaries for the most vulnerable functional roles. The decline of large mobile predators may be partly compensated for only by wilderness areas through larval dispersal and adult spillover into fished areas^39^.

## Methods

### Study sites

**Figure 1:**
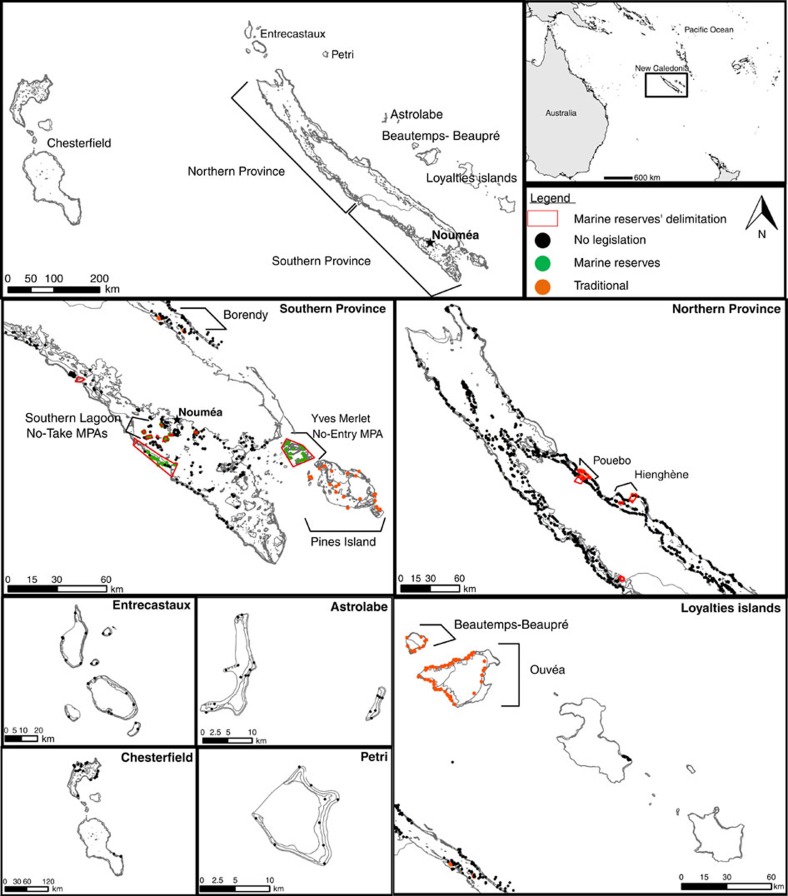
Location of study areas in New Caledonia. Sites were located at the outer reef, back reef, lagoon reef or fringing reef depending on the locations and configurations of the reefs. Reef fishes and habitats were surveyed at each transect. Transects (1833) were performed between 1986 and 2013 in areas without legislation (black dots), in marine reserves (green dots) and in traditionally managed areas (orange dots).

**Figure 2:**
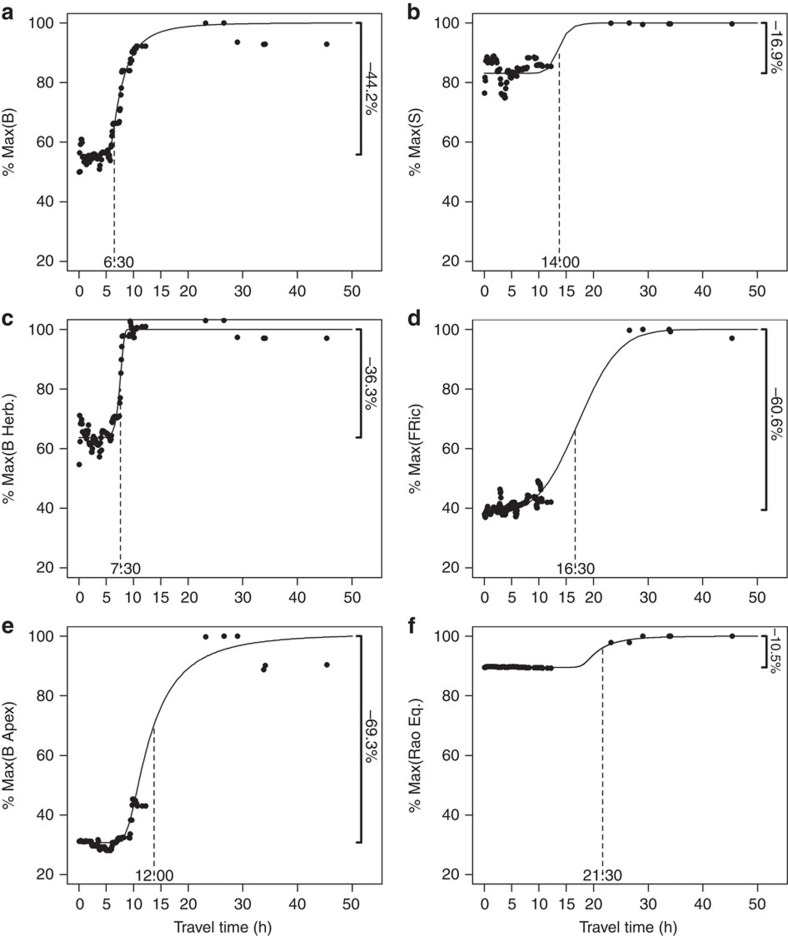
Partial dependence plots for six fish community metrics along a gradient of travel time from the market. (**a**) Total biomass (B), (**b**) species density (S), (**c**) herbivores biomass (B Herb.), (**d**) functional richness (FRic), (**e**) apex predator biomass (B APEX) and (**f**) biomass-weighed functional diversity (Rao Entropy). Fish community metrics were predicted using eight to nine environmental and human explanatory variables depending of the community metrics (Methods, Supplementary Table 1 and Supplementary Figs 1 and 2). The *y* axis is the percentage of variation from the maximum value for each community aspect. The decrease between this maximum value (asymptote) and the bottom line is expressed as a percentage. Inflection point estimates (in hours) are indicated with a dotted vertical line.

**Figure 3:**
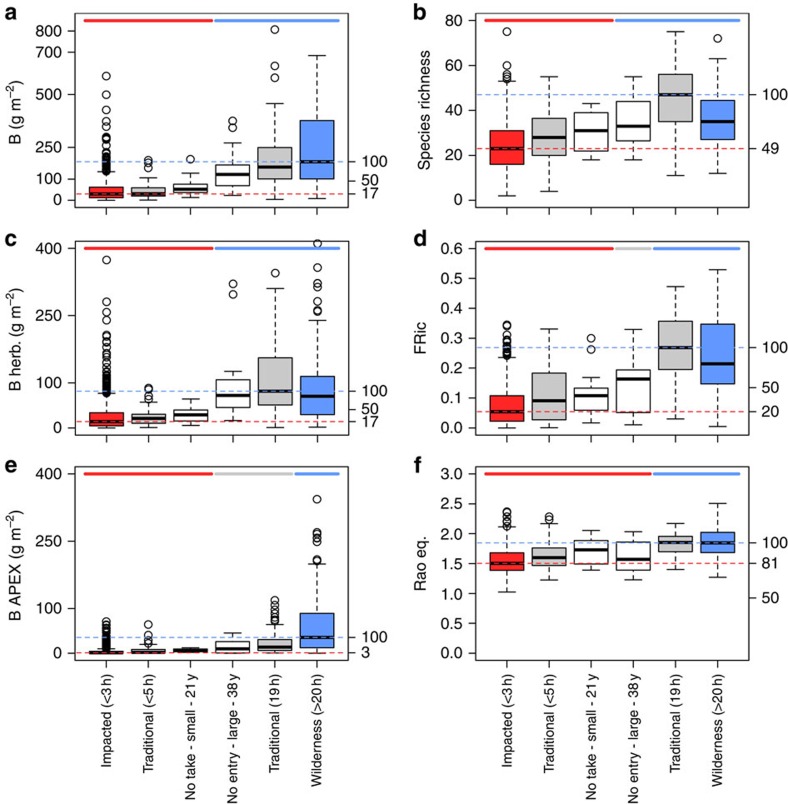
Human influence on six fish community metrics for six different categories of areas. Boxplot distributions of (**a**) total biomass (B), (**b**) species richness (S), (**c**) herbivores biomass (B Herb.), (**d**) functional richness (FRic), (**e**) apex predator biomass (B APEX) and (**f**) biomass-weighed functional diversity (Rao Entropy) for fish communities in fished areas at <3 h from the main market (red), in the small no-take marine reserves at <1 h from the main market, in the large, old and no-entry marine reserve at <2 h from market, in traditionally managed areas at <5 h travel time, isolated traditionally managed areas (19 h travel time) and in areas located at >20 h from the main market (blue) (Supplementary Fig. 4). Biomasses were log transformed. The median values across fish communities sampled in wilderness areas (>20 h) for these metrics were considered as benchmarks and were set at the maximum possible value (100%) (right *y* axis). Coloured horizontal bars indicate the clusters of areas after *a post-hoc* Kruskal–Wallis test, the red bars indicate areas with fish community metrics similar to those in exploited areas (<3 h), blue bars indicate areas with fish community metrics similar to those in wilderness areas (>20 h) and grey bars indicate areas with similar intermediate values for fish community metrics (Supplementary Table 3).

**Figure 4:**
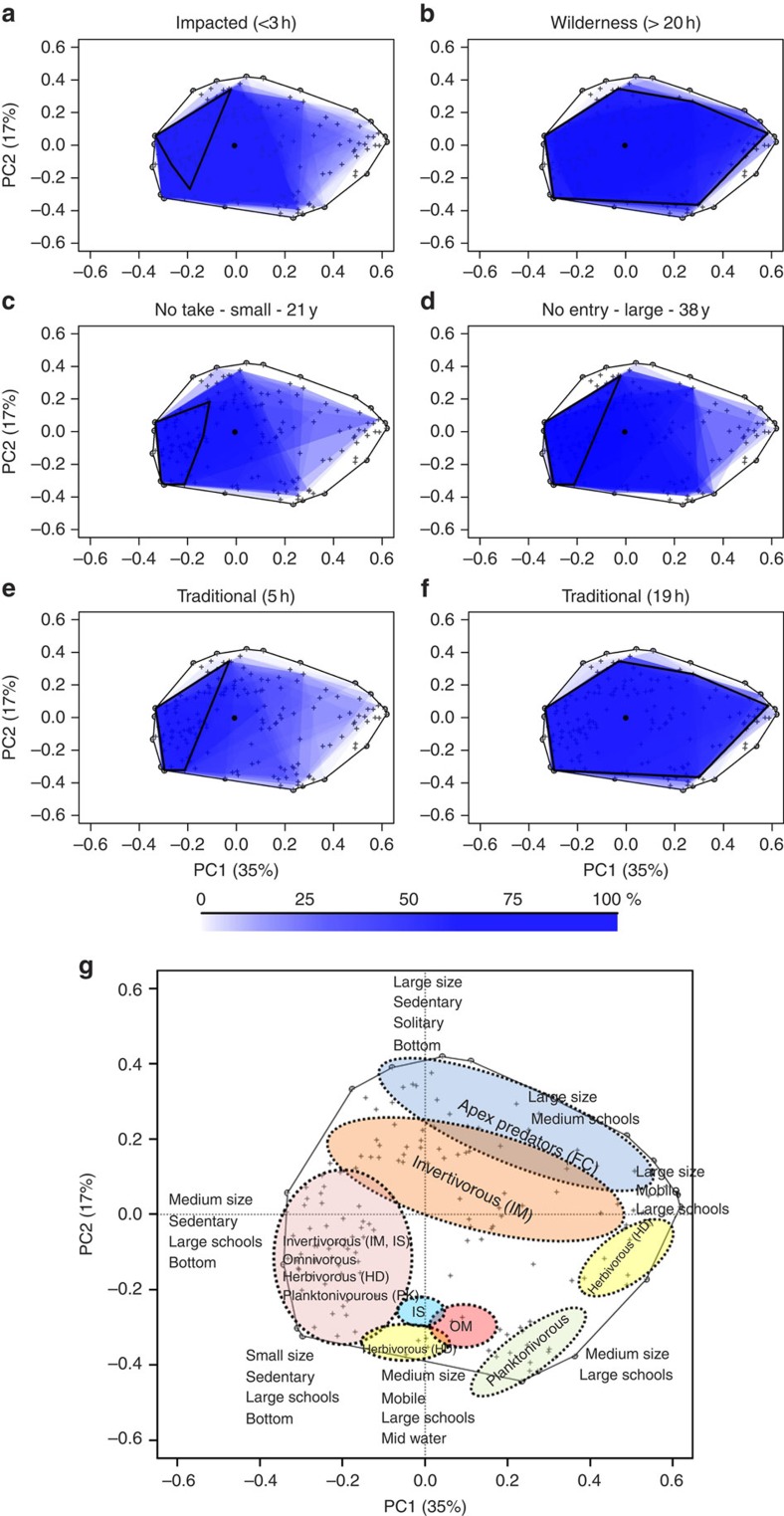
Breadth of functional roles played by fish communities in different categories of areas. Functional space filled by fish communities in (**a**) areas at <3 h of travel from the market, (**b**) areas at >20 h from the market, (**c**) no-take small marine reserves, (**d**) no-entry, old and large marine reserve, (**e**) traditionally managed areas at <5 h from the market and (**f**) traditionally managed areas at 19 h from the market. The 50% contour lines represent the functional space filled by fishes in >50% of the communities for a given category of area. The ecological meaning of the functional space is given in **g**. The percentage of the total functional space filled by fish communities is given in Supplementary Table 4 and the details on the traits are provided in Supplementary Fig. 6.

New Caledonia is located in the South Pacific, ∼1,200 km off eastern Australia (Fig. 1). This archipelago comprises a main high island, the ‘Grande Terre', the Loyalty Islands and several smaller islands. The ‘Grande Terre' is surrounded by one of the largest barrier reef systems in the world (24,000 km^2^)^40^. One third of the human population lives along the shore of the southwest lagoon where Noumea, the main city (∼98,000 people) is located (www.isee.nc). Human density is very high around Noumea (2,135 people per km^2^) compared with the remainder of the country (<5 people per km^2^) (www.isee.nc).

### Coral reef management in New Caledonia

As MPAs considered here are all no take, we adopt the terminology of ‘marine reserve' for no-take MPAs (category I IUCN) to avoid confusion with areas where management allows fishing^3^. Marine reserves managed by provincial administrations cover ∼42,000 ha^41^. Many other areas along the coast are managed traditionally (taboo areas) by local tribes and are coined as traditionally managed areas.

Fishing and collection of resources are prohibited in marine reserves (no take), but access is open, except in the ‘Yves Merlet' marine reserve where only scientists with special authorization are allowed (IUCN category Ia)^41^.

Three kinds of managed areas were included in the study (Fig. 1): (i) the large, old and no-entry marine reserve ‘Yves Merlet' (38 years old, 17,500 ha); (ii) small, no-take marine reserves (‘Lagon Sud', 21 years old, <700 ha); and (iii) six traditionally managed areas.

### Survey methodology

Reef fishes and the associated coral reef habitats were surveyed from 1986 to 2013 across New Caledonia, spanning ∼5° latitude and ∼9.5° longitude. Data were collected along 1,833 underwater visual transects located from highly populated (2,135 people Per km^2^) to isolated and uninhabited sites. There was no temporal replication.

The main reef types (biotope) were included: (1) sheltered coastal reef, (2) lagoon reef, (3) inner barrier reef and (4) outer reef. For each reef, transects were performed on both the reef flat and slope, when feasible. Transects were oriented parallel to the depth contour between 0 and 15 m^42^.

Distance-sampling underwater visual census (D-UVC)^43^ technique was used to survey finfishes along 50-m-long transects in selected sites. Briefly, this method involved two divers, where each diver recorded the species, abundance, body length and distance perpendicular to the transect line of each fish or group of fish, while swimming slowly down the line^43^.

The minimum distance at which individuals are recorded is potentially influenced by the level of human pressure in the zone (repulsion in exploited sites and attraction in remote sites). With fixed transect width, fish density can be underestimated due to repulsion in exploited sites and overestimated in remote sites^44^,^45^,^46^,^47^.

In wilderness areas, the median distance per transect at which individuals (>10 cm) were observed is 4 m and even 3 m for the most isolated areas. In the meantime, the median distance in fished areas close to the market could reach up to 7 m (Supplementary Fig. 7a,c).

Individuals <10 cm represented 8.5% of the total individuals and this density was constant along the human gradient (Supplementary Fig. 7b).

To decrease the bias due to diver attraction and repulsion, D-UVC data sets were truncated at a distance of 7 m on each side of transects. This incorporated ∼95% of sighted commercially important species and all top predators, allowing for the calculation of abundance, biomass and diversity over a 700-m^2^ area (2 sides × 7 m width × 50 m long) (Supplementary Fig. 7e,f).

### Species density and biomass

Sharks and rays were removed from the main species list due to the difficulties in assessing their occurrence and abundance with D-UVC^48^.

This study focused on 352 species of commercially important teleost fish species (33 families) (Supplementary Table 6). Top predators (69 species) were characterized by trophic level >4 (ref. 49). Trophic levels were obtained from FishBase for each species^50^. Herbivorous species (89 species) were split into herbivore–detritivore and macroalgal feeders, and invertebrate sessile feeders.

Species richness was estimated as the number of species per 700 m^2^ transect.

The biomass of individual fishes was estimated using the allometric length–weight conversion: *W*=*a*TL^*b*^, where parameters *a* and *b* are species-specific constants, TL is the individual total fork length in cm and *W* is the weight in *g*^51^. Biomass was log-transformed for statistical analyses.

### Fish functional traits

We characterized fishes using six functional traits linked to diet and feeding behaviour^52^: (1) maximum size, (2) diet, (3) mobility, (4) position over the reef, (5) activity and (6) gregariousness^52^,^53^. Fish size was coded using six ordered categories: 0–7, 7.1–15, 15.1–30, 30.1–50, 50.1–80 and >80 cm. Mobility was coded using three ordered categories: sedentary (including territorial species), mobile within a reef and mobile between reefs. Activity period was coded using three ordered categories: diurnal, both diurnal and nocturnal, and nocturnal. Schooling was coded using five ordered categories: solitary, pairing or living in small (3–20 individuals), medium (20–50 individuals) or large (>50 individuals) groups. Vertical position in the water column was coded using three ordered categories: benthic, bentho-pelagic and pelagic. Diet was characterized based on main items consumed by each species, which resulted into seven trophic categories: herbivorous–detritivorous (that is, fish feeding on turf or filamentous algae and/or undefined organic material), macroalgal herbivore (that is, fishes eating large fleshy algae and/or seagrass), invertivores targeting sessile invertebrates (that is, corals, sponges and ascidians), invertivores targeting mobile invertebrate (that is, benthic species such as crustaceans), planktivorous (that is, fishes eating small organisms in the water column), piscivores (including fishes and cephalopods) and omnivores (that is, fishes for which both vegetal and animal material are important in their diet)^52^,^54^.

### Fish functional space

Pairwise functional distances between species were computed using the Gower distance, which allows mixing different types of variables, while giving them equal weight^55^. A Principal Coordinates Analysis was then performed using this functional distance matrix and the first four principal axes were retained to build a multidimensional functional space^31^,^56^.

### Functional diversity

We used two functional diversity indices that are known to be linked to community assembly and ecosystem processes^57^:

Functional Richness for each community was measured as the volume inside the convex hull occupied by species of the community^31^,^56^. Functional Richness was computed using the presence/absence data only. It represents the breadth of trait ranges in the community and can be considered as a proxy for the diversity of roles.

Biomass-weighed functional diversity^58^ (Rao's quadratic entropy):


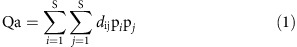


where *d*_*ij*_ is the functional distance between the *i*-th and the *j*-th species (Gower distance) and *p*_*i*_ and *p*_*j*_ are the relative local biomass of the *i*-th and the *j*-th species, respectively.

The Rao entropy considers both biomass distribution among species and pairwise functional distances between species. It measures the extent of dissimilarity in a community between two randomly chosen species from a community^57^,^59^,^60^. The Rao entropy is not trivially related to species richness, as it is both dependent on the biomass of species and their functional distinctness, which may decrease or increase with the addition of species^61^. If a new species entering the community is highly functionally dissimilar but has a low biomass, the Rao entropy will not increase substantially. This is a conservative measure of functional diversity, with the main ecological assumption being that functional groups with low biomass should not contribute substantially to ecosystem processes whatever their functional traits.

We applied the correction derived from equivalent numbers of species^60^:


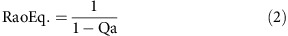


The equivalent number is the number of maximally functionally distinct species having equal biomass, which produces maximal entropy^59^,^60^.

The Rao equivalent number of species is an unbiased measure of the Rao entropy.

### Explanatory variables

We examined the relationships between the six fish biodiversity indices and key environmental and human variables hypothesized to influence the conditions of reef fish communities in New Caledonia at multiple spatial scales.

Environmental variables: (i) reef area (km^2^) in a buffer radius (km) (Global Distribution of Coral reefs 2010), collected from the United Nations Environment Programme World Conservation Monitoring Centre (www.unep-wcmc.org) as a measure of habitat availability and connectivity^62^,^63^. Three buffers were calculated to take into account the ecological and evolutionary scales of connectivity. We used 3 and 30 km buffers^63^ as proxies of ecological connectivity and 600 km buffer as proxy of evolutionary connectivity^64^. The Pearson correlation coefficients between the three reef surface variables range from 0.1 to 0.3; (ii) mean depth (m); (iii) live coral cover (%); (iv) reef type (fringing, lagoon, inner barrier reef and outer reef); (v) slope (slope or reef flat area to consider habitat stratification and interactions with species^42^); (vi) longitude and latitude; (vii) weekly average SST (1998–2008 in a 5-km pixel) to consider the geographic extent and the temperature gradient in the study area (temperature AVHRR (Advanced Very High Resolution Radiometer); http://oceanwatch.pifsc.noaa.gov/). For each UVC transect, we calculated the temperature within that pixel; (viii) island type^65^ with three categories: high island (island without lagoon, including tectonically uplifted reefs, such as Lifou), low island (island with a large lagoon such as ‘La Grande Terre') and atoll (no island, except reef islands, which are islands that are created by the accumulation of reef sediments).

Human variables: we used minimum travel time to market (h) as a the main driver of fishing pressure^16^,^17^ to model fish biodiversity and biomass. The major market in New Caledonia is in the capital city of Noumea.

The travel time to Noumea indicates the proximity of the market and is a proxy for fish market demand in New Caledonia^66^. Fish merchants drive 4–5 h each week to the north of New Caledonia to buy reef fishes (∼3 tons) and invertebrates from fishers in local tribes (Pers. Com.). Fishes and invertebrates are then sold at the Noumea market and in local supermarkets and restaurants.

Travel time by road between Noumea and the closest village from each transect were retrieved using Google APIs application in R (XML and RCurl packages). For islands, the travel time is the commercial boat trip duration if the islands are linked by commercial routes. For transects not located on ‘La Grande Terre' and more than 50 km away from the first inhabited area, the travel time was computed directly between Noumea and the transect using the average speed of a fishing boat (10 nm h^−1^). Travel times between the first inhabited areas and the transects were computed using the average speed of a small boat (15 nm h^−1^). The total travel time between the transects and Noumea is the sum of the travel time between the transects and the first inhabited areas, and the travel time between this inhabited area and Noumea.

We also considered human population, through the human density in a 50-km radius around each transect. As the transects were sampled between 1986 and 2013, we rebuilt demography of New Caledonia from four population census years (1989, 1996, 2004 and 2009) for the main cities and smaller known populated locations (villages and tribes) (www.isee.nc). Transects were classified regarding the closest census: (i) 1989 census: 1986–1992; (ii) 1996 census: 1995–1998; (iii) 2004 census: 2001–2005; and (iv) 2009 census: 2008–2013.

Collinearity exists between environmental and human variables. To avoid confounding effects in the model, we tested relationships between explanatory variables. Both travel time and human population were positively correlated with latitude (Travel Time: *r*-Pearson=0.47, *P*<0.0001; Human density: *r*-Pearson=0.67, *P*<0.0001). Latitude was significantly correlated with both reef area in a 600-km buffer (*r*-Pearson=0.5604, *P<*0.0001) and SST (*r*-Pearson=0.72, *P<*0.0001).

The number of inhabitants within a 50-km buffer (log) was correlated with travel time (*r*-Pearson=−0.87, *P<*0.0001) (Supplementary Fig. 3). To avoid confounding effects of explanatory variables due to their correlation, we only kept SST and travel time to market for analyses. The human indices based on the number of inhabitants within a buffer may give the false impression that in transects with values close to zero there is no human impact, whereas travel time to market is more integrative with a distal human influence providing a more continuous assessment (no threshold effect due to the buffer) (Supplementary Fig. 3).

Overall, we selected ten environmental and one human variables as follows: (i) mean depth (m), (ii) live coral cover (%), (iii) reef type, (iv) the area of reef slope in a 3-km radius, (v) the area of reef slope in a 30-km radius, (vi) the area of reef slope in a 600-km radius, (vii) SST (°C), (viii) longitude, (ix) the slope and (x) the type of island, and (xi) travel time (h) to market.

The final set of variables included into the models follow a 3-step process: (i) choice of 11 environmental and 2 human variables hypothesized to influence the conditions of reef fish communities in New Caledonia at multiple spatial scales; (ii) removal of correlated variables to avoid confounding effects in the model. From this step, ten environmental and one human variables were selected; (iii) simplification procedure recommended by Elith *et al*.^18^ to remove variables having <5% contributions to the model. Between eight and nine explanatory variables were included in the final simplified models depending of the biodiversity and biomass index.

### Statistical analyses

To decouple the relative effects of environment, humans and management on biodiversity, we used BRTs, which are able to cope with strongly interacting factors and nonlinear relationships^18^. A low CV predictive deviance, hence a high correlation coefficient from CV (CV *R*^2^) and a sufficient number of trees (*N* trees) (>1,000) are essential criteria to select a good BRT model^18^. To address these criteria, we chose the optimal combination of tree complexity (tc), learning rate (lr) and bag fraction (bf) as the one minimizing the out-of-bag estimates of error rate with an *N* tree>1,000^18^. In BRTs, contributions of each explanatory variable (%) are the proportion of each variable selected to split the data among all the trees, weighted by the squared improvement to the model as a result of each split, and averaged over all trees^18^. The variables with the highest percentage contributions are the most important variables contributing to the model. We removed explanatory variables with <5% contributions to the model^18^ that explained the difference between the set of variables in the ‘full model' (without simplifications) and the simplified model.

All BRTs were built in R (R Development Core Team 2011 version R version 2.15.2) using the gbm package version 1.6-3.1 and custom code available online^18^. To test whether we missed some key variables, which are trivially related to the geographic placement of transects, we performed spatial autocorrelation analyses using the Moran's index for the residuals of modelled biodiversity and biomass components (results shown in Supplementary Fig. 8).

### The ‘no-legislation' model

To estimate the relative and pure effect of isolation on each fish community aspect, we first fitted a ‘no-legislation' BRT model using the 11 explanatory variables (live coral, depth, biotope, slope, type of island and reef area in a 3, 30 or 600 km radius, SST, longitude and travel time to market) that may influence the ecological values of reef fish communities and only the 1,626 transects in areas where no management actions, whether from tribes or provinces, are applied.

### Human marginal effect

The marginal effect of travel time to market on fish biodiversity components was estimated after accounting for the average effects of all other variables in the ‘no-legislation' model^18^.

To characterize the relationship between each ecological aspect and human influence, we compared four candidate models: a null model, a sigmoid model (five-parameter logistic model^67^), a hyperbolic model and a power model. The Akaike information criterion was used to compare models, whereby lower-valued Akaike information criterion scores provided support for one model over another.

The linear model was not selected, as the infinite growth of biodiversity or biomass metrics is not realistic as it is bounded by the regional species pool and system carrying capacity. Raw relationships between travel time and fish biodiversity and biomass components are shown in Supplementary Fig. 9.

### The ‘management' model

To extract the ‘pure' effect of management on biodiversity components, a ‘Management' explanatory variable was constructed using the following classes of human influence (areas with no management at <3, 3–5, 5–10, 10–20 and >20 h travel time from Noumea market) and four types of management (no-take small marine reserve, no-entry large marine reserve, traditional <5 h and traditional 19 h).

For each fish community aspect, a BRT model using 8 environmental and the categorized ‘Management' explanatory variables was built on the complete database (1,833 transects) and the marginal effect of ‘Management' was estimated after accounting for the average effects of all other variables in the model.

We then compared the levels of fitted biomass and biodiversity among marine reserves, traditionally managed areas and areas located at <3 h and at >20 h of travel time using the Kruskal–Wallis non-parametric test and its associated *post-hoc* pair-wise comparisons (letter-based representation^68^), as well as the non-parametric effect size, which is the ratio of the z score from the pairwise Wilcoxon test divided by the square-root of the number *n* of transects (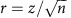)^69^. An effect size <0.2 is considered small, <0.5 is moderate and >0.8 is large^70^.

### Data availability

The data that support the findings of this study are available from L.V. (laurent.vigliola@ird.fr) upon request.

## Additional information

**How to cite this article:** D'agata, S. *et al*. Marine reserves lag behind wilderness in the conservation of key functional roles. *Nat. Commun.* 7:12000 doi: 10.1038/ncomms12000 (2016).

## Supplementary Material

Supplementary Information Supplementary Figures 1-9 and Supplementary Tables 1-6
