# Supplementary material for: Marine reserves lag behind wilderness in the conservation of key functional roles
Source: Nat Commun. 2016 Jun 29;7:12000. doi: 10.1038/ncomms12000 (PMC4931279; doi:10.1038/ncomms12000)
Supplement: Supplementary Information — Supplementary Figures 1-9 and Supplementary Tables 1-6 [file ncomms12000-s1.pdf]

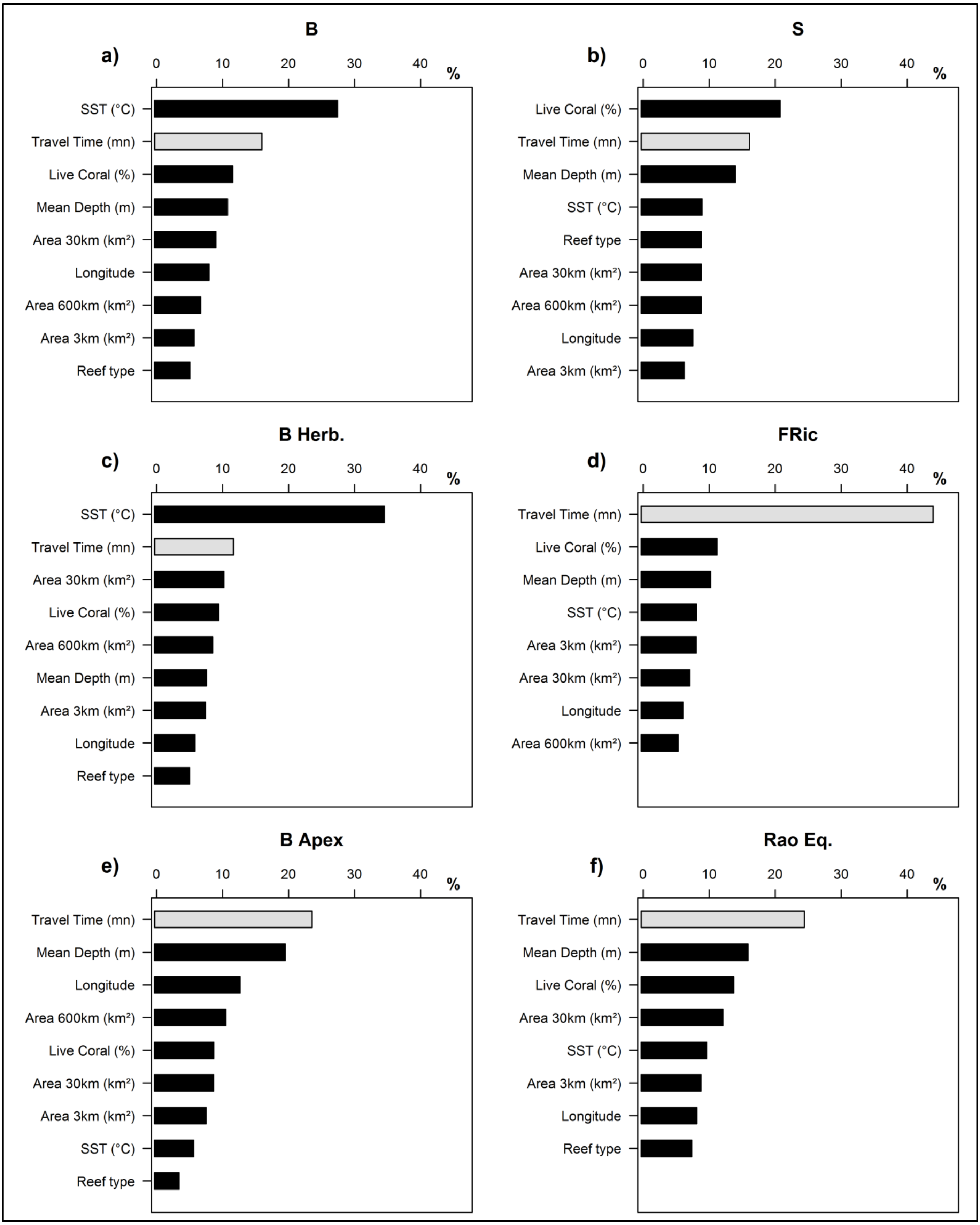

Supplementary Figure 1. Contributions of explanatory variables for the simplified no-legislation model. The contributions of each explanatory variable (%) (no legislation) from simplified BRT models are given for a) the Total Biomass ($g.m^{-2}$), b) Species Richness (Number of species per transect), c) Herbivores biomass (g/m^{-2}), d) Functional Richness (FRic), e) Apex biomass (g/m^{-2}) and f) Biomass-weighed functional diversity (Rao Entropy) converted to equivalent number of species. The variable "Travel Time to market (h)" is highlighted in light grey.

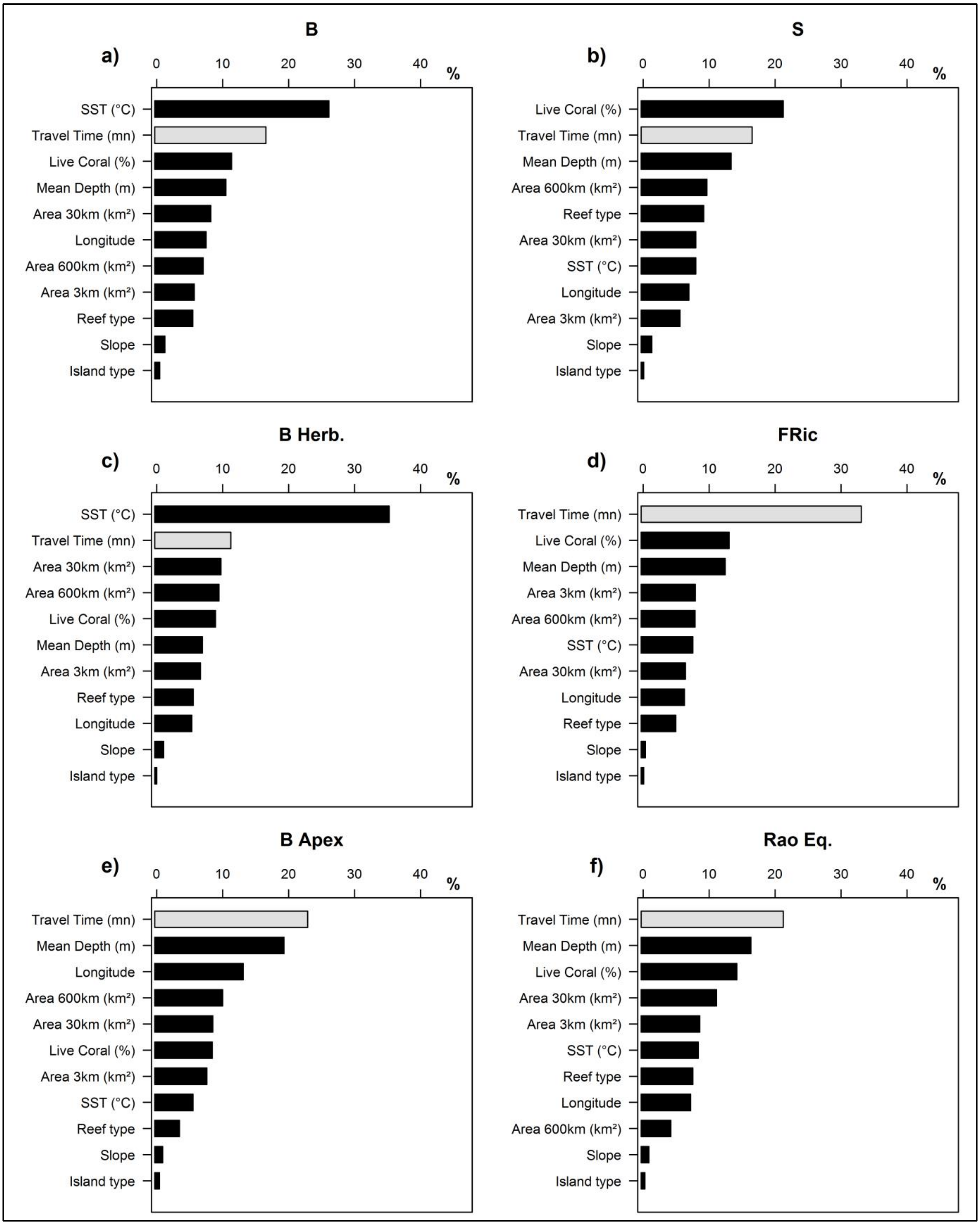

Supplementary Figure 2. Contributions of explanatory variables for the full no-legislation model.
The contributions of each explanatory variable (%) (no legislation) from full BRT models are given for a) the Total Biomass ($g.m^{-2}$), b) Species Richness (Number of species per transect), c) Herbivores biomass ($g.m^{-2}$), d) Functional Richness (FRic), e) Apex biomass ($g.m^{-2}$) and f) Biomass-weighed functional diversity (Rao Entropy) converted to equivalent number of species. The variable "Travel Time to market (h)" is highlighted in light grey.

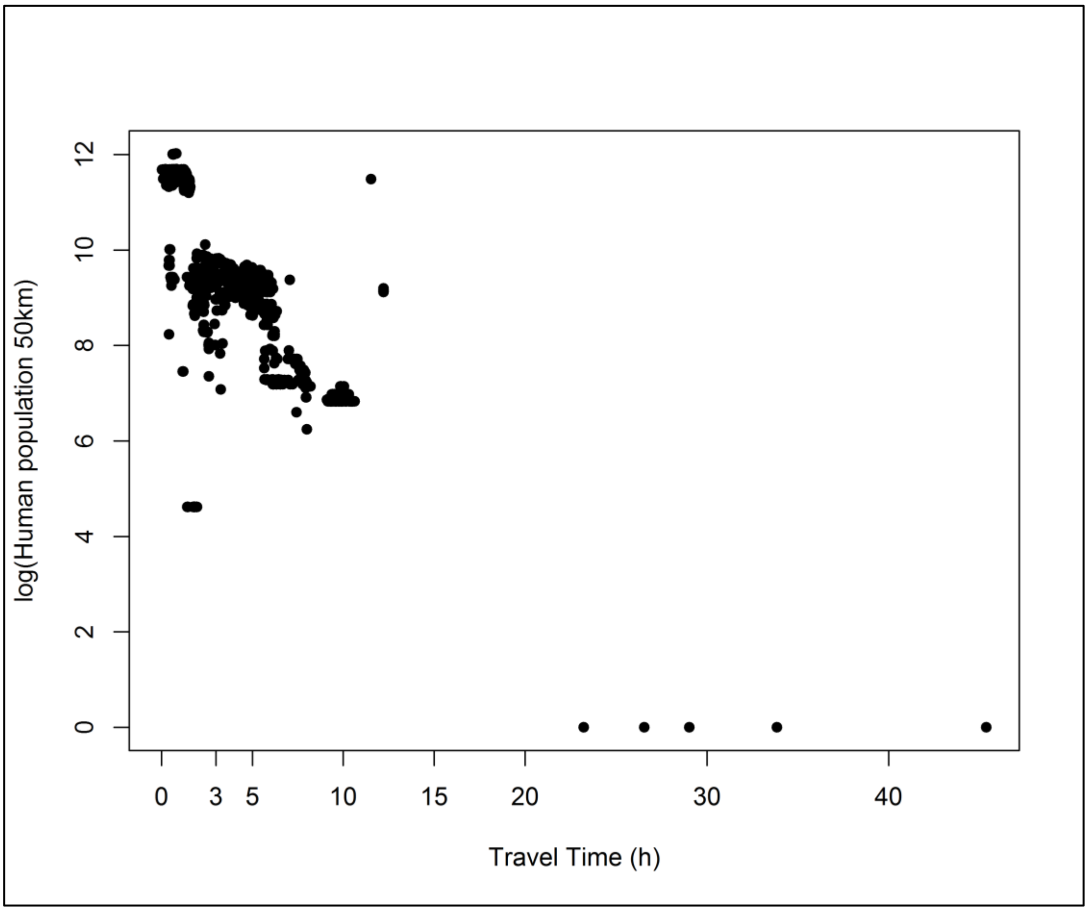

Supplementary Figure 3. Relationship between local human population and travel time.
Relationship between the log of the local human population in a 50km buffer around each transect and the travel time to market (Noumea).

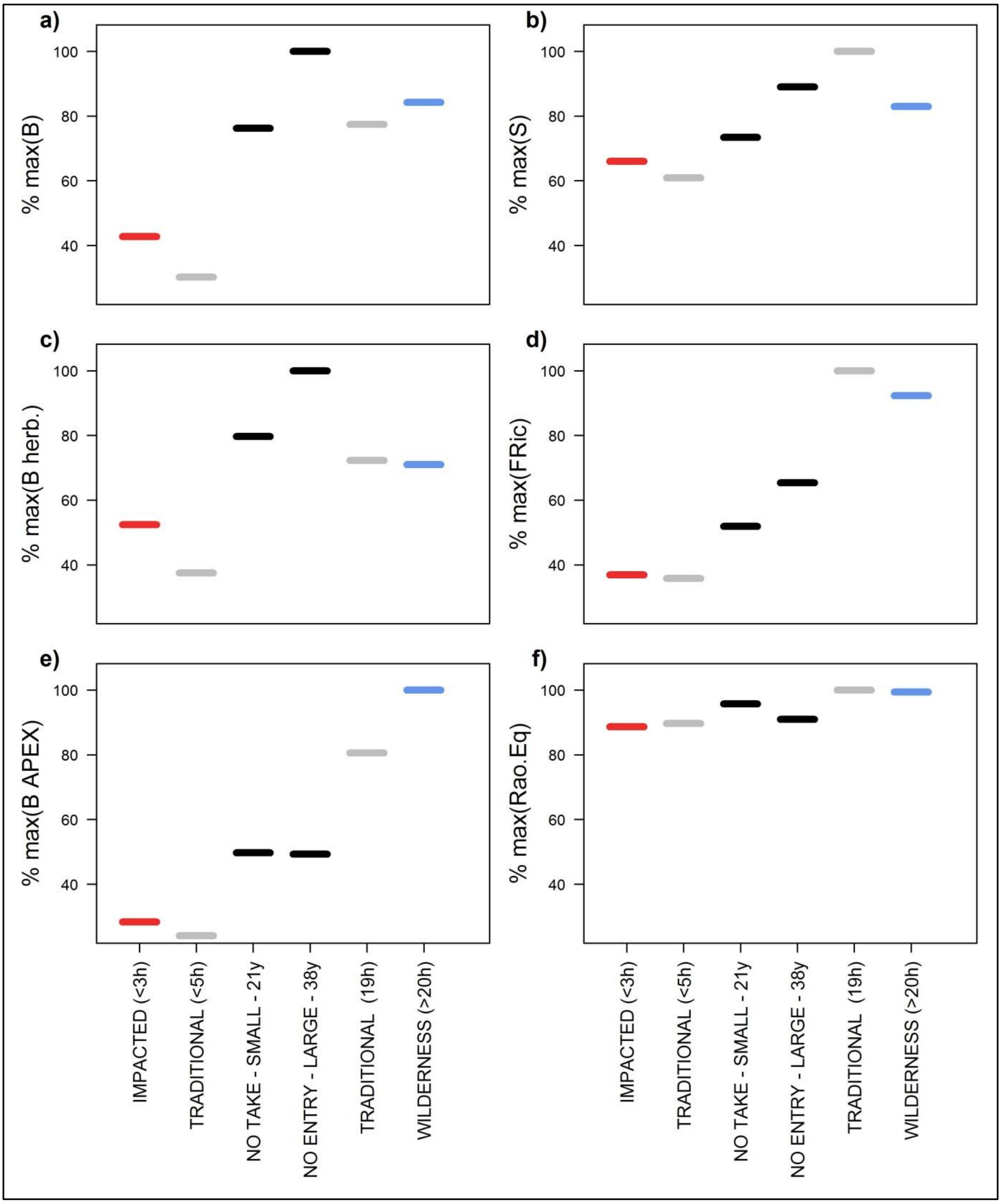

Supplementary Figure 4. Partial dependence plots of biomass and biodiversity indices for different categories of areas.

Fitted variations were predicted using 8 environmental explanatory variables and the "management" as the human variables as predictors in the BRT models. The left y-axis is the percentage of variation from the maximum value for each community aspect. The percentage of the maximum value is independent of the range and the unit of each index and thus comparable between indices.

The fitted levels of fish community aspects for different managements: i) exploited areas (< 3h) (red), ii) the no-take small MPAs (black), iii) the no-entry large MPA (black), iv) the traditionally managed areas < 5 hours travel time (grey), v) the traditionally managed areas at 19 hours travel time (grey) and vi) the wilderness areas (> 20h) (blue).

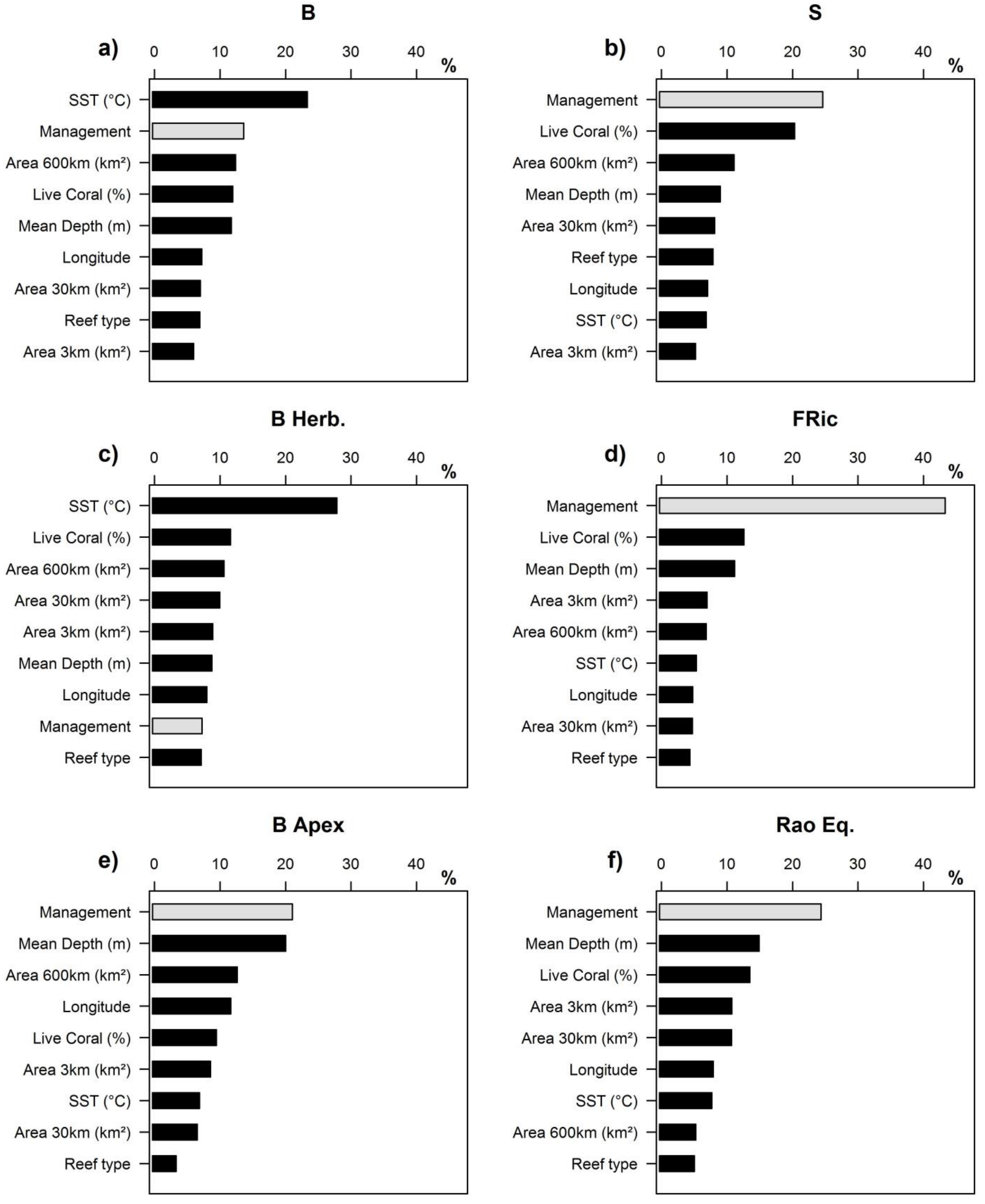

Supplementary Figure 5. Contributions of explanatory variables for different fish community aspects using the simplified "Management" model.
The contributions of each explanatory variable (%) from "Management" simplified BRT models are given for a) the Total Biomass ($g.m^{-2}$), b) Species Density (Number of species per transect), c) Herbivores biomass ($g.m^{-2}$), d) Functional Richness (FRic), e) Apex biomass ($g.m^{-2}$) and f) Biomass-weighted functional diversity (Rao entropy) converted to equivalent number of species. The variable "Management" is highlighted in light grey.

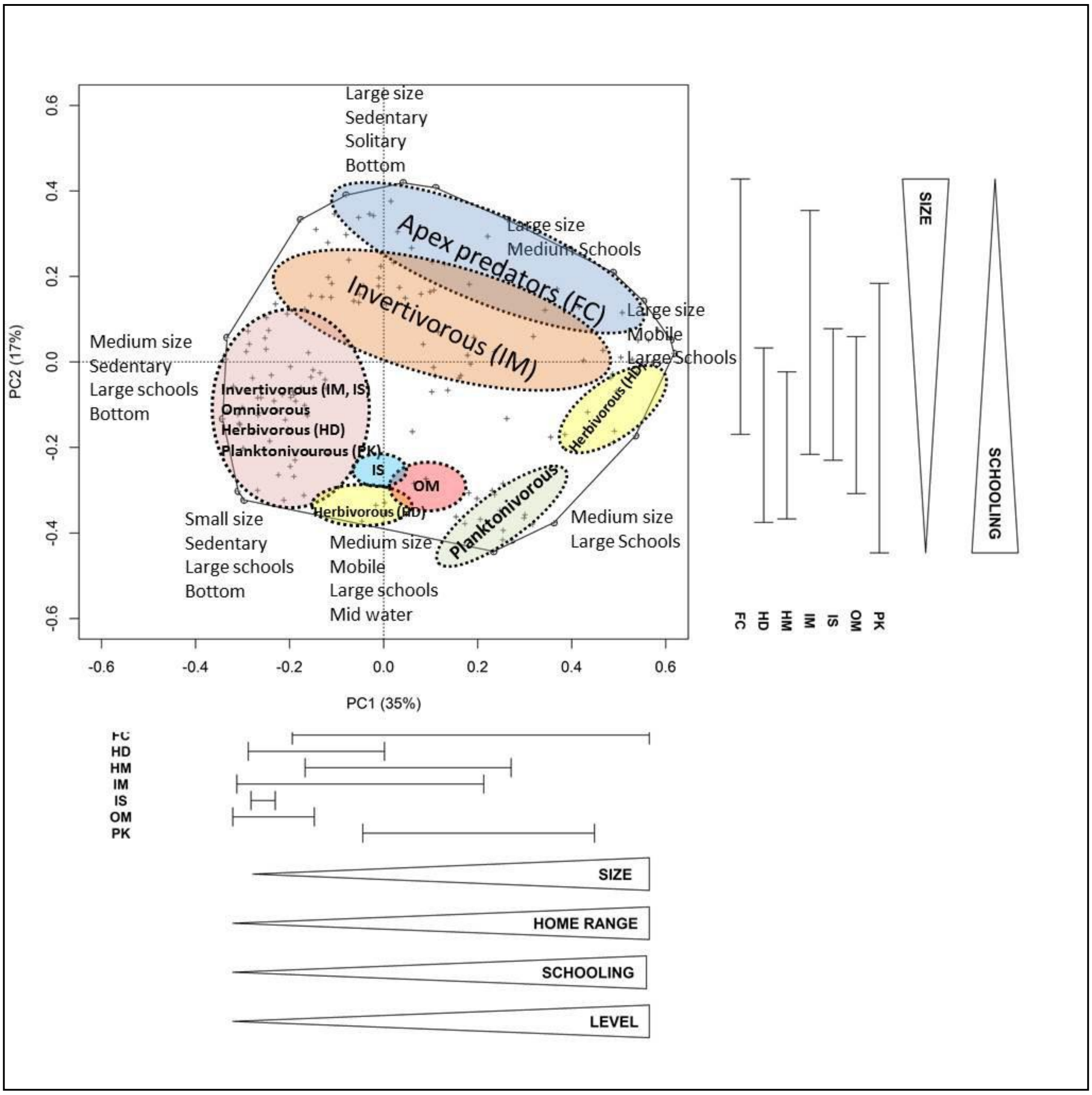

Supplementary Figure 6. Functional space with PCoAs axes characterization.

Ecological characterization of the functional space with five most important functional traits related to PCoA axes 1 and 2: "Size" of fish, "Home-range" or the mobility of fish, 3) "Schooling" or the gregariousness of fish species, 4) "Level" or the vertical position in the water column and 5) the "Diet" with 7 categories (HD: herbivorous-detritivorous, HM:macroalgal herbivorous, IS: invertivorous targeting sessile invertebrates, IM: invertivorous targeting mobile invertebrate, PK: planktivorous, FC: piscivorous, and OM: omnivorous).

The range of coordinates along PCoA axes for the different modalities of ordered qualitative functional traits were used to define the trait modalities characterizing PCoA axes. For the "Size", "Home range", "Schooling" and "Level" traits, the larger part of arrows indicate the highest values of the modalities. For the "Diet" trait, the range of values is indicated for each modality

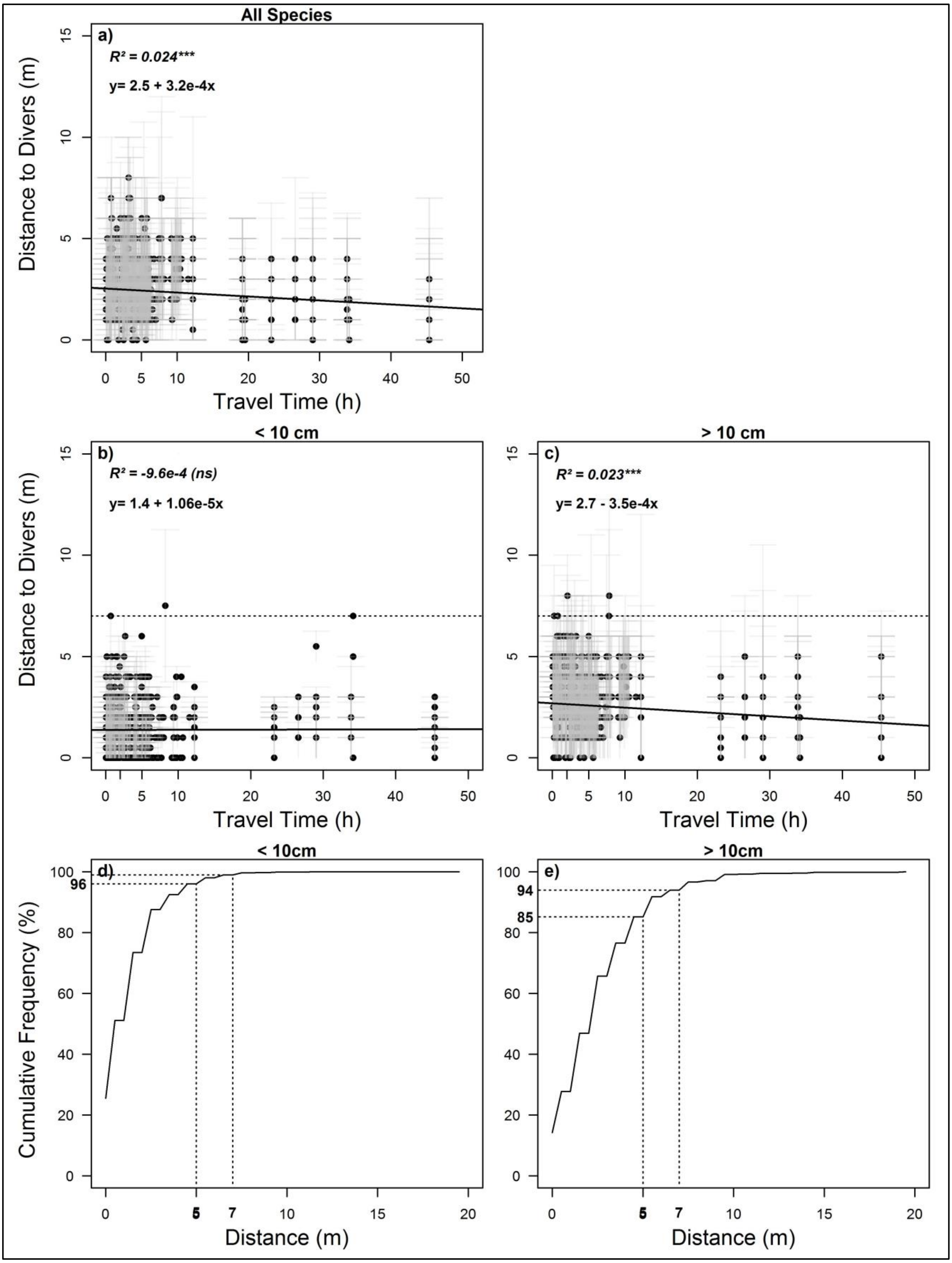

Supplementary Figure 7. Estimation of fish distance orthogonal to divers along a gradient of travel time (h) and cumulative frequency of fish occurrence.

Linear relationships between the median distance (dots) of fish individuals to divers (m) for each transect and travel time (h) to the main market for a) all fish, b) fish <10 cm and c) fish >10 cm. Grey bars indicate 25th and 75th percentiles.

Cumulative frequency of fish occurrences along the travel time to market (h) gradient for d) fish < 10cm and e) fish > 10cm. The percentage of the total number of fish individuals for 5m and 7m distance to divers are indicated in bold along the left y-axis. (ns), p>0.05; * $p \leq 0.05$; ** $p \leq 0.01$; *** $p \leq 0.001$; *** $p \leq 0.0001$.

At a very small scale we may have some autocorrelation and thus pseudoreplication which inflates p-values.

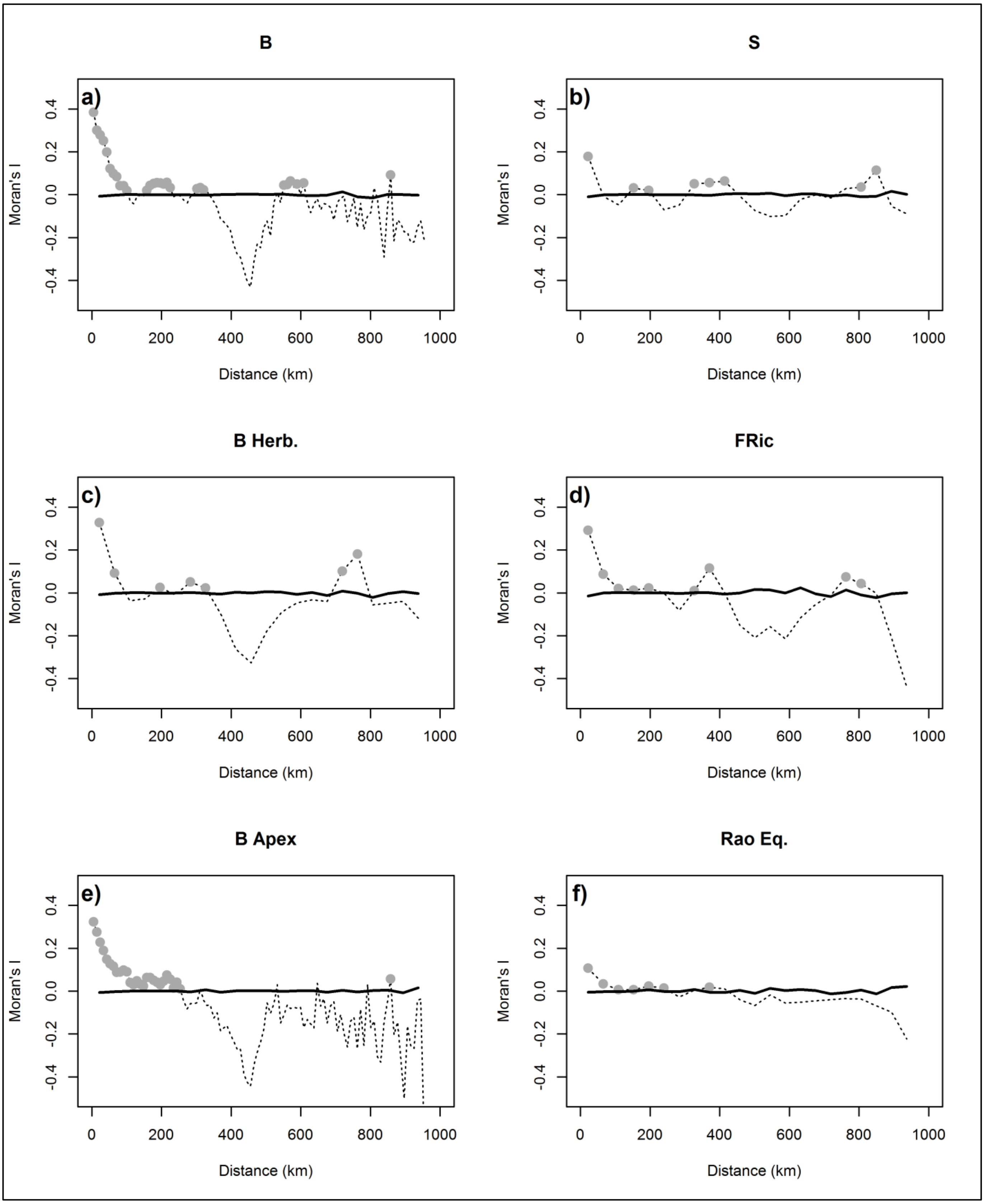

Supplementary Figure 8. Residuals spatial autocorrelation for each modelled aspect of fish community structure.

Autocorrelation in raw data (back dash line) and residual autocorrelation (bold lines) for a) the Total Biomass (g.m-²), b) Species Density (Number of species per transect), c) Herbivores biomass (g.m-²), d) Functional Richness (FRic), e) Apex biomass (g.m-²) and f) Biomass-weighted functional diversity (Rao entropy).

Grey dots indicate significant autocorrelation (p.val < 0.05).

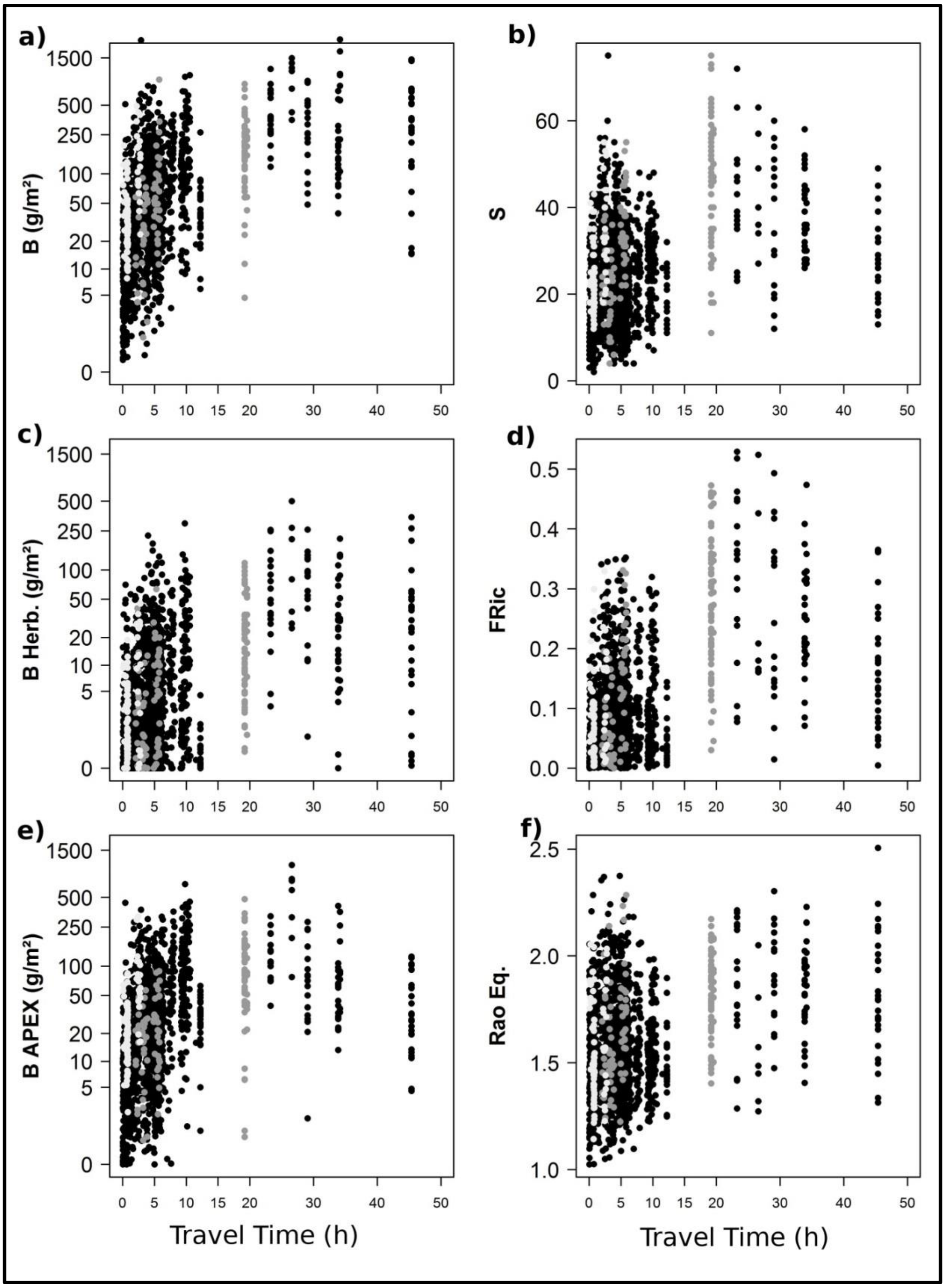

Supplementary Figure 9. Raw data for each aspect of fish community structure along the gradient of travel time from the market.

Raw relationships between the travel time to market (h) and a) the Total Biomass (g.m-²), b) Species Density (Number of species per transect), c) Herbivores biomass (g.m-²), d) Functional Richness (FRic), e) Apex predator biomass (g.m-²) and f) Biomass-weighed functional diversity (Rao Entropy).

In black are raw data for "no-managed areas", in dark gray for "traditional areas" and in light grey for marine protected areas.

Supplementary Table 1. Parameters and performance of simplified BRT models.
Parameters and performance of simplified BRT models for Biomass of commercial fish (B), Biomass of apex predators (B APEX) and biomass of herbivores (B HERB.), Species density (S), Rao Equivalent of number of species (Rao Eq.) and Functional Richness (FRic). lr is the learning rate, tc is the tree complexity, N.trees is the number of optimal trees, R^2Tr is the correlation coefficient based on training dataset, R^2CV is the correlation coefficient from the k-fold cross-validation procedure with n folds (nf) equal to 20 and bag fraction (bf), SE R^2_{CV} is the standard error. D^2 is the cross-validated proportion of the total deviance explained.

Index	lr	tc	N.trees	R^2_{Tr}	R^2_{CV}	SE R^2_{CV}	bf	D^2
B	0.004	7	2900	0.814	0.676	0.014	0.7	45.23
B APEX	0.005	5	1850	0.751	0.634	0.017	0.7	48.89
B HERB.	0.005	8	2500	0.843	0.699	0.012	0.7	40.39
S	0.004	8	3500	0.841	0.661	0.016	0.7	43.20
FRic	0.003	5	1800	0.782	0.611	0.024	0.7	37.95
Rao EQ.	0.003	7	1200	0.561	0.396	0.025	0.7	15.32

Supplemental Table 2. Directions of the relationships between environmental variables and biomass and biodiversity aspect for the No-Legislation model and the Management model.
(+ : positive relationship; - : negative relationship; X : flat relationship; Black: No influence).

Explanatory variables		Live Coral (%)	Mean Depth (m)	Area 3km (km^2)	Area 30km (km^2)	Area 600km (km^2)	Longitude	SST (°C)	Reef type				VertZone		Island Type		
									Back	Outer	Intermediate	Fringing	Slope	Flat	Low	High	Atoll
No Legislation	B	+	+	x	+	+	-	+	+	+	+	-	■	■	■	■	■
	B Herb	+	+	+	x	+	x	+	+	+	+	-	■	■	■	■	■
	B Apex	+	+	x	+	+	-	x	+	+	+	-	■	■	■	■	■
	S	+	+	x	+	+	+	+	+	+	+	-	■	■	■	■	■
	Fric	+	+	x	-	+	-	+	■	■	■	■	■	■	■	■	■
	Rao Eq.	+	+	x	-	■	-	+	+	+		-	■	■	■	■	■
Management	B	+	+	x	x	+	-	+	+	+	+	-	■	■	■	■	■
	B Herb	+	+	+	+	+	x	+	+	+	+	-	■	■	■	■	■
	B Apex	+	+	x	x	+	-	x	+	+	-	-	■	■	■	■	■
	S	+	+	x	+	+	x	+	+	+	+	-	■	■	■	■	■
	Fric	+	+	x	x	+	x	x	+	+	+	-	■	■	■	■	■
	Rao Eq.	+	+	+	-	■	x	x	+	+	+	-	■	■	■	■	■

Supplementary Table 3. Model selection results for candidate models
Models in bold have the lowest AIC. The ΔAIC values indicate distance between the top two ranked models. ΔAIC values > 2 indicate substantial support for the lower-values model.

INDEX	Constant	Sigmoid	Hyperbolic	Power	ΔAIC
B	-3477.0	**-7039.8**	-4426.1	-4416.1	2613.7
B APEX	-3950.4	**-6503.6**	-4499.5	-4870.5	1633.1
B Herb.	-3575.4	**-6578.9**	-4215.8	-3762.3	2363.1
S	3890.1	**3629.4**	4490.4	3889.9	260.5
FRic	-8681.156	**-10989.5**	-4908.235	-8866.269	2123.2
Rao Eq.	-7929.529	**-12630.83**	-8272.521	-7945.571	4358.3

Supplementary Table 4. Wilcoxon and non-parametric effect size pairwise comparisons between management levels.

Wilcoxon (W test and p-value) and non-parametric effect size (r) pairwise comparisons between the 6 levels of management for: i) exploited areas ("< 3h"), ii) the no-take small MPAs, iii) the no-entry large MPA, iv) the small traditionally managed areas under 5 hours of travel time, v) the traditionally managed areas at 19 hours of travel time and vi) the wilderness areas ("> 20h)".The results of the post-hoc Kruskal-Wallis test are indicated for each community aspect with letters. The results of the pairwise Wilcoxon tests (W value and its p-value) are indicated. The non-parametric effect size is the ratio of the z score from the Wilcoxon test divided by the square-root of the number n of transect ($r = z/\sqrt{n}$). (ns), $p>0.05$; * $p \leq 0.05$; ** $p \leq 0.01$; *** $p \leq 0.001$; *** $p \leq 0.0001$.

At a very small scale we may have some autocorrelation and thus pseudoreplication which inflates p-values.

B Herb.

		< 3h	*NO TAKE SMALL*	*NO ENTRY LARGE*	*TRADITIONAL < 5h*	*TRADITIONAL ~ 19h*	*>20h*
		a	ab	bc	a	c	bc
< 3h	a		W= 6991**<br>r= -0.108	W= 9993***<br>r= -0.226	W= 21083 (ns)<br>r= -0.001	W= 30885****<br>r= -0.365	W= 48280****<br>r= -0.400
NO TAKE SMALL	ab	W= 7060**<br>r= -0.112		W= 306 ***<br>r= -0.6541	W= 425*<br>r= -0.214	W= 951***<br>r= -0.522	W= 1382***<br>r= -0.348
NO ENTRY LARGE	bc	W= 9752****<br>r= -0.214	W= 277**<br>r= -0.502		W= 168***<br>r= -0.554	W= 703 (ns)<br>r= -0.146	W= 996 (ns)<br>r= -0.060
TRADITIONAL < 5h	a	W= 19399 (ns)<br>r= -0.012	W= 371*<br>r= -0.275	W= 183***<br>r= -0.537		W= 3392****<br>r= -0.644	W= 5247****<br>r= -0.567
TRADITIONAL ~ 19h	c	W= 31861****<br>r= -0.392	W= 950***<br>r= -0.520	W= 776*<br>r= -0.220	W= 3526****<br>r= -0.702		W= 2750 (ns)<br>r= -0.011
>20h	c	W= 51974****<br>r= -0.481	W= 1540***<br>r= -0.453	W= 1317*<br>r= -0.227	W= 5737****<br>r= -0.697	W= 3415 (ns)<br>r= -0.1162	

S

		< 3h	*NO TAKE SMALL*	*NO ENTRY LARGE*	*TRADITIONAL < 5h*	*TRADITIONAL ~ 19h*	*>20h*
		a	ab	bcd	ab	d	c
< 3h	a		W= 7138**<br>r= -0.116	W= 8858***<br>r= -0.1677	W= 22352 (ns)<br>r= -1e-04	W= 31883***<br>r= -0.393	W= 44403****<br>r= -0.324
NO TAKE SMALL	bcd	W= 822***<br>r= -0.174		W= 227*<br>r= -0.278	W= 500 (ns)<br>r= -0.135	W= 923***<br>r= -0.487	W= 1135*<br>r= -0.190
NO ENTRY LARGE	abc	W= 8101**<br>r= -0.129	W= 203 (ns)<br>r= -0.184		W= 430*<br>r= -0.262	W= 899**<br>r= -0.355	W= 1017.5 (ns)<br>r= -0.069
TRADITIONAL < 5h	abc	W= 20673 (ns)<br>r= -0.002	W= 371*<br>r= -0.275	W= 447*<br>r= -0.244		W= 3240***<br>r= -0.578	W= 4177***<br>r= -0.288
TRADITIONAL ~ 19h	cd	W= 30908****<br>r= -0.366	W= 810**<br>r= -0.348	W= 779*<br>r= -0.223	W= 3125 ***<br>r= -0.528		W= 1821 (ns)<br>r= 0
>20h	d	W= 52554****<br>r= -0.494	W= 1540***<br>r= -0.453	W= 1565***<br>r= -0.378	W= 5518****<br>r= -0.639	W= 4247***<br>r= -0.323	

Rao Eq.

		< 3h	*NO TAKE SMALL*	*NO ENTRY LARGE*	*TRADITIONAL < 5h*	*TRADITIONAL ~ 19h*	*>20h*
		a	ab	a	a	b	b
< 3h	a		W= 7419**<br>r= -0.130	W= 6885 (ns)<br>r= -0.070	W= 22482 (ns)<br>r= -1e-04	W= 30061****<br>r= -0.3415	W= 46696****<br>r= -0.371
NO TAKE SMALL	ab	W= 7048**<br>r= -0.123		W= 144 (ns)<br>r= -0.0296	W= 443*<br>r= -0.194	W= 757*<br>r= -0.2839	W= 1177*<br>r= -0.215
NO ENTRY LARGE	bc	W= 8101.5***<br>r= -0.142	W= 208 (ns)<br>r= -0.202		W= 635 (ns)<br>r= -0.070	W= 902**<br>r= -0.358	W= 1420**<br>r= -0.289
TRADITIONAL < 5h	ab	W= 21566 (ns)<br>r= -1e-04	W= 516 (ns)<br>r= -0.112	W= 510 (ns)<br>r= -0.172		W= 2971***<br>r= -0.461	W= 4564***<br>r= -0.388
TRADITIONAL ~ 19h	d	W= 32908.5****<br>r= -0.452	W= 988***<br>r= -0.568	W= 1013***<br>r= -0.485	W= 3310***<br>r= -0.632		W= 3178 (ns)<br>r= -0.067
>20h	cd	W= 50409.5****<br>r= -0.483	W= 1467***<br>r= -0.404	W= 1458***<br>r= -0.312	W= 4903***<br>r= -0.496	W= 2591 (ns)<br>r= -0.004	

Supplementary Table 5. Percentage of regional functional volume filled by the six different categories of areas. The percentage of the regional functional volume for 352 commercial reef fish species have been computed as the ratio between the functional volume shared by 50% of the transects divided by the regional functional volume. n is the number of transect per location.

Site	% Regional Functional Volume<br>50%	n
< 3h	12.1	360
NO TAKE - SMALL	12.1	27
NO ENTRY - LARGE	15.7	21
TRADITIONAL <5h	15.2	56
TRADITIONAL ~ 19h	76.7	56
> 20h	76.7	100

Supplementary Table 6. Families (33) and genus (97) list for the 352 species

Family	Genus
Acanthuridae	Acanthurus
Acanthuridae	Ctenochaetus
Acanthuridae	Naso
Acanthuridae	Paracanthurus
Acanthuridae	Zebrasoma
Aulostomidae	Aulostomus
Balistidae	Balistapus
Balistidae	Balistoides
Balistidae	Melichthys
Balistidae	Odonus
Balistidae	Pseudobalistes
Balistidae	Rhinecanthus
Balistidae	Sufflamen
Balistidae	Xanthichthys
Caesionidae	Caesio
Caesionidae	Pterocaesio
Carangidae	Alectis
Carangidae	Atule
Carangidae	Carangoides
Carangidae	Caranx
Carangidae	Elagatis
Carangidae	Gnathanodon
Carangidae	Scomberoides
Carangidae	Trachinotus
Chanidae	Chanos
Diodontidae	Diodon
Echeneidae	Echeneis
Ephippidae	Platax
Fistulariidae	Fistularia
Haemulidae	Diagramma
Haemulidae	Plectorhinchus
Haemulidae	Pomadasys
Holocentridae	Myripristis
Holocentridae	Neoniphon
Holocentridae	Sargocentron
Kyphosidae	Kyphosus
Labridae	Bodianus
Labridae	Cheilinus
Labridae	Choerodon
Labridae	Coris
Labridae	Epibulus
Labridae	Gomphosus
Labridae	Hemigymnus
Labridae	Oxycheilinus
Lethrinidae	Gnathodentex
Lethrinidae	Gymnocranius
Lethrinidae	Lethrinus
Lethrinidae	Monotaxis
Lutjanidae	Aphareus
Lutjanidae	Aprion
Lutjanidae	Lutjanus
Lutjanidae	Macolor
Lutjanidae	Symphorus

Family	Genus
Monacanthidae	Aluterus
Monacanthidae	Amanses
Monacanthidae	Cantherhines
Mugilidae	Upeneus
Mugilidae	Valamugil
Mullidae	Mulloidichthys
Mullidae	Parupeneus
Muraenidae	Gymnothorax
Nemipteridae	Scolopsis
Ostraciidae	Ostracion
Pomacanthidae	Centropyge
Pomacanthidae	Genicanthus
Pomacanthidae	Pomacanthus
Pomacanthidae	Pygoplites
Priacanthidae	Priacanthus
Scaridae	Bolbometopon
Scaridae	Calotomus
Scaridae	Cetoscarus
Scaridae	Chlorurus
Scaridae	Hipposcarus
Scaridae	Leptoscarus
Scaridae	Scarus
Scombridae	Euthynnus
Scombridae	Grammatorcynus
Scombridae	Gymnosarda
Scombridae	Rastrelliger
Scombridae	Sarda
Scombridae	Scomberomorus
Scorpenidae	Pterois
Serranidae	Aethaloperca
Serranidae	Anyperodon
Serranidae	Cephalopholis
Serranidae	Cromileptes
Serranidae	Diploprion
Serranidae	Epinephelus
Serranidae	Gracila
Serranidae	Plectropomus
Serranidae	Variola
Siganidae	Siganus
Sparidae	Acanthopagrus
Sphyraenidae	Sphyraena
Tetraodontidae	Arothron
Tetraodontidae	Canthigaster
Zanclidae	Zanclus
